# Behavioral and neurophysiological indices of the racial bias modulation after virtual embodiment in other-race body

**DOI:** 10.1016/j.isci.2023.108085

**Published:** 2023-09-28

**Authors:** Maria Pyasik, Alice Mado Proverbio, Lorenzo Pia

**Affiliations:** 1 SAMBA (SpAtial, Motor and Bodily Awareness) Research Group, Department of Psychology, University of Turin, 10124 Turin, Italy; 2 Cognitive Electrophysiology Lab, Department of Psychology, University of Milano-Bicocca, 20126 Milan, Italy; 3 NIT (Neuroscience Institute of Turin), 10124 Turin, Italy

**Keywords:** Social interaction, Neuroscience, Behavioral neuroscience

## Abstract

Racial bias—nonconscious behavioral inclinations against people of other ethnic groups—heavily contributes to inequality and discrimination. Immersive virtual reality (IVR) can reduce implicit racial bias through the feeling of owning (embodying) a virtual body of a different “race”; however, it has been demonstrated only behaviorally for the implicit attitudes. Here, we investigated the implicit (racial IAT) and the neurophysiological (the N400 component of the event-related potentials for verbal stimuli that violated negative racial stereotypes) correlates of the embodiment-induced reduction of the implicit racial bias. After embodying a Black avatar, Caucasian participants had reduced implicit racial bias (IAT) but both groups showed the typical N400. This is the first evidence to suggest that virtual embodiment affects the evaluative component of the implicit biases but not the neurophysiological index of their cognitive component (i.e., stereotyping). This can inform interventions that promote inclusivity through the implicit/indirect procedures, such as embodiment.

## Highlights

• Behavioral and neurophysiological effects of embodiment on racial bias were studied
• Implicit racial bias decreased after embodiment in a racially out-group avatar
• Out-group embodiment did not affect N400 for incongruences in racial stereotypes
• Out-group embodiment differentially affects distinct components of racial bias

## Graphical abstract

**Figure:**
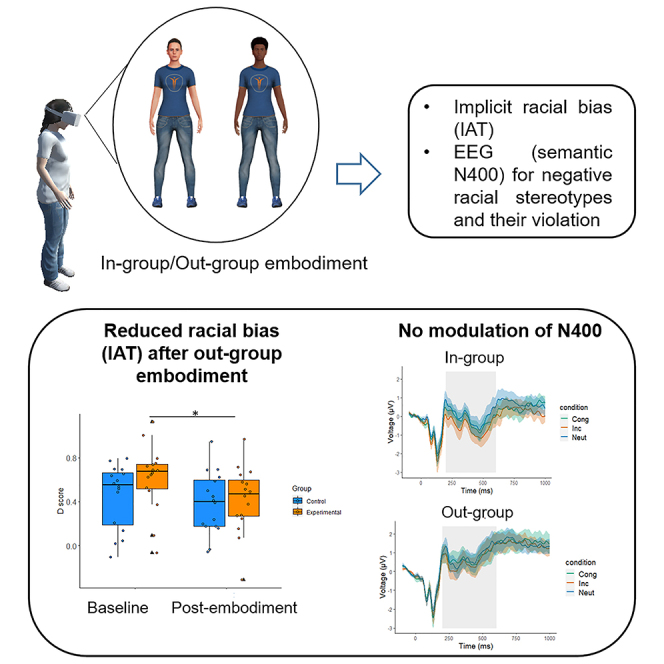

## Introduction

In-group biases—behavioral inclinations against certain people or groups that are rooted in the negative stereotypes about those groups—are thought to be one of the core mechanisms of discrimination.^1^ They can be present between any social groups, e.g., those of different race, gender, or age, and manifest explicitly or be implicit, with the latter being more problematic, as it can affect the behavior even when a person explicitly denies being biased against a certain group.^2^ One profound example of bias in Western societies is the one against all non-white people—the racial bias that spreads to multiple concepts and behaviors, thus perpetuating social inequality.^3^ Considering the strong negative effects of the implicit racial bias for society, it is necessary to explore effective ways to mitigate it and to understand in-depth the mechanisms by which such mitigation can occur. Indeed, it is known that commonly used explicit interventions aimed at reducing the racial bias are short-lived and often contradictory.^1^^,^^4^ This can be explained by the implicit (nonconscious) nature of the racial bias and other social biases: it cannot be overtly influenced if its presence is consciously denied.^2^ Therefore, it would be worth exploring an approach that would be able to act at the nonconscious level, thus overcoming this problem.

One promising tool for investigating this topic is immersive virtual reality (IVR). In particular, such technique allows inducing a temporary illusion of owning a body of an out-group (i.e., other race) virtual character. In this procedure, participant sees a virtual body (avatar) from the first-person perspective (1PP) and therefore perceives that body as her own, consequently perceiving the external environment from the perspective of that body. This illusory experience, called “embodiment,”^5^ stems from the flexible nature of bodily self-awareness, in particular, body ownership (the feeling that one’s body belongs to oneself^6^). Embodiment is based on the integration of synchronous multisensory information and has several constraints: to be embodied, the object has to be human-like; it has to be seen in an anatomically plausible position (congruently with the body or directly from the 1PP); and synchronous multimodal stimulation has to be delivered (participant’s body and the fake/virtual body are touched synchronously or participant’s movements need to be synchronous with the movements of the fake/virtual body).^7^

Crucially, embodiment occurs independently of the appearance of the virtual body, i.e., it is strong even when the avatar’s appearance is drastically different from participant’s own,^8^ which makes it a possible tool for influencing the appearance-based stereotypes (such as racism or sexism) and the related biases in a covert way. Indeed, when participants observe the world from the perspective of a differently looking body that they perceive as their own, or control the movements of that body, they do not receive any explicit instructions concerning the body appearance. Yet, owning an out-group body has been shown to affect the implicit in-group biases. In the domain of the racial bias, several studies showed that implicit racial bias was temporarily reduced after brief embodiment of a black avatar by white participants.^9^^,^^10^^,^^11^^,^^12^^,^^13^

Despite being promising in terms of both basic scientific knowledge and practical applications, the results of these studies are based on one primary outcome measure, namely, the implicit associations test, IAT.^14^ The IAT measures individual statistical associations between categories, and stronger associations between pairs of concepts evoke faster responses. For example, by presenting different combinations of images of Black and White faces paired with “negative” and “positive” words, one can quantify the strength of the associations that are representative of the racial bias (e.g., White-positive, Black-negative). The IAT is therefore based on semantic associations, and the measures (reaction times and accuracy of categorization) are nonconscious because they probe the semantic link between the concepts (i.e., their closeness in the semantic matrix). Even though the IAT has been shown to have predictive power for implicit in-group biases,^15^ it reflects only one aspect of the phenomenon at the behavioral level, namely, its evaluative/emotional component, without providing any evidence about the cognitive component of the racial bias, i.e., the stereotyping.^16^^,^^17^ Therefore, the currently existing data on the embodiment-induced reduction of the implicit racial bias are incomplete at the behavioral level and are entirely lacking any kind of neural correlate, without which this potentially relevant effect remains poorly understood.

To fill this gap, in the present study, we aimed at probing the neurophysiological signatures of the effects of virtual embodiment on the implicit racial bias. The neural/neurophysiological mechanisms of the racial bias have been investigated in neuroimaging studies that described differential patterns of neural activation in relation to prejudice and stereotyping, i.e., the emotional/evaluative and the cognitive processes that underlie the racial bias, respectively.^18^^,^^19^ In particular, it has been shown that stereotyping was related to the activation of the anterior temporal lobes and the medial and dorsolateral prefrontal cortices, whereas prejudice was subserved by the activation of the amygdala, orbital frontal cortex, insula, striatum, and medial prefrontal cortex^16^; see also^17^^,^^20^ for review. In turn, studies that employed electroencephalography (EEG) to investigate the neurophysiological correlates of the implicit racial bias pinpointed the N400 component of the event-related potentials (ERPs) as a possible neurophysiological index related to stereotyping, i.e., to the cognitive component of the racial bias. The N400 is a negative wave deflection peaking around 200–600 ms after the stimulus onset over centroparietal electrodes, which is typically related to processing semantically incongruent information and the difficulty of its integration with the previous knowledge,^21^ included the violation of stereotypes related to the world knowledge.^22^^,^^23^^,^^24^ Importantly, an increased N400 was observed for stimuli (sentences) that violated racial stereotypes,^25^ which makes this ERP component a possible neurophysiological proxy of the implicit racial bias. Moreover, a reduction of N400 was demonstrated after the explicit modulation of the racial bias/stereotypes by exposure to socially positive information about people of different ethnicities.^26^ Therefore, in the present EEG study, we focused on the N400 as the neurophysiological index of the implicit racial bias.

**Figure 1:**
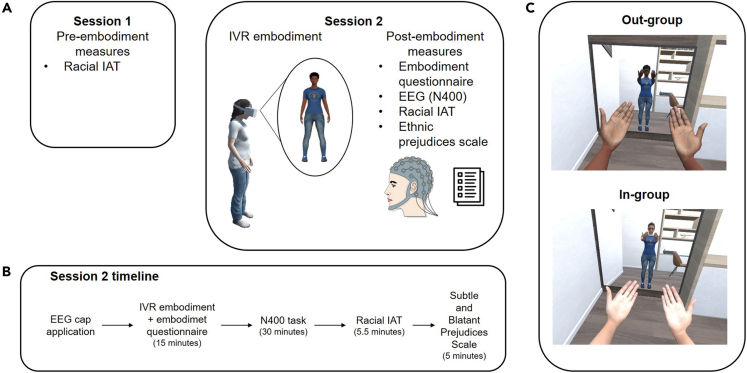
Experimental setup and procedures (A) Experimental design and tasks. (B) Timeline of session 2 with durations of each task. (C) Embodiment procedure in the two experimental conditions: participant observes the avatar (out-group or in-group, according to the skin color) from the 1PP and as a mirror reflection, and participant’s movements are synchronized with those of the avatar.

In order to achieve this aim, we modulated the implicit racial bias through the virtual embodiment of a racially out-group avatar (in the experimental group), which was compared with the embodiment of a racially in-group avatar (in the control group) and measured the effects of such modulation at the neurophysiological level via the EEG. After virtual embodiment, the N400 was registered in a task that consisted of reading sentences that were congruent, incongruent, or neutral with respect to negative racial stereotypes. Along with the N400, two other ERP components that could be affected by the implicit bias modulation were analyzed: P300 and frontal positivity that reflect the activation of expectations about the stimuli and the emotional valence of the stimuli, respectively.^25^ In addition, the changes at the level of implicit associations (racial IAT administered before and after the embodiment) were evaluated to compare the effects of the out-group embodiment with the existing findings. Finally, the explicit racial bias was measured via a questionnaire as a control measure, as it has been shown that the explicit racial bias is not modulated by the virtual embodiment (e.g.,^10^); see Figure 1 for a summary of the experimental setup and procedures.

We hypothesized that both the implicit (IAT) and the neurophysiological (N400) indices of the racial bias would decrease after the embodiment of an out-group avatar but not after the embodiment of an in-group avatar. In line with,^26^ the P300 and the frontal positivity were not expected to change depending on the embodiment condition but were hypothesized to differ according to different stimulus types. In particular, larger P300 was expected for the stimuli congruent with the negative racial stereotypes, as they would match the expectations created by the first part of the sentence, and larger frontal positivity was expected for the congruent and incongruent stimuli compared with the neutral ones.

## Results

### Embodiment questionnaire

To evaluate whether the virtual embodiment was comparable for the racially out-group and in-group avatars, the ratings in the illusion statements were compared between groups with Mann-Whitney U test; with groups, each illusion statement was compared with the corresponding control statement with Wilcoxon signed rank test (see STAR methods for details).

**Figure 2:**
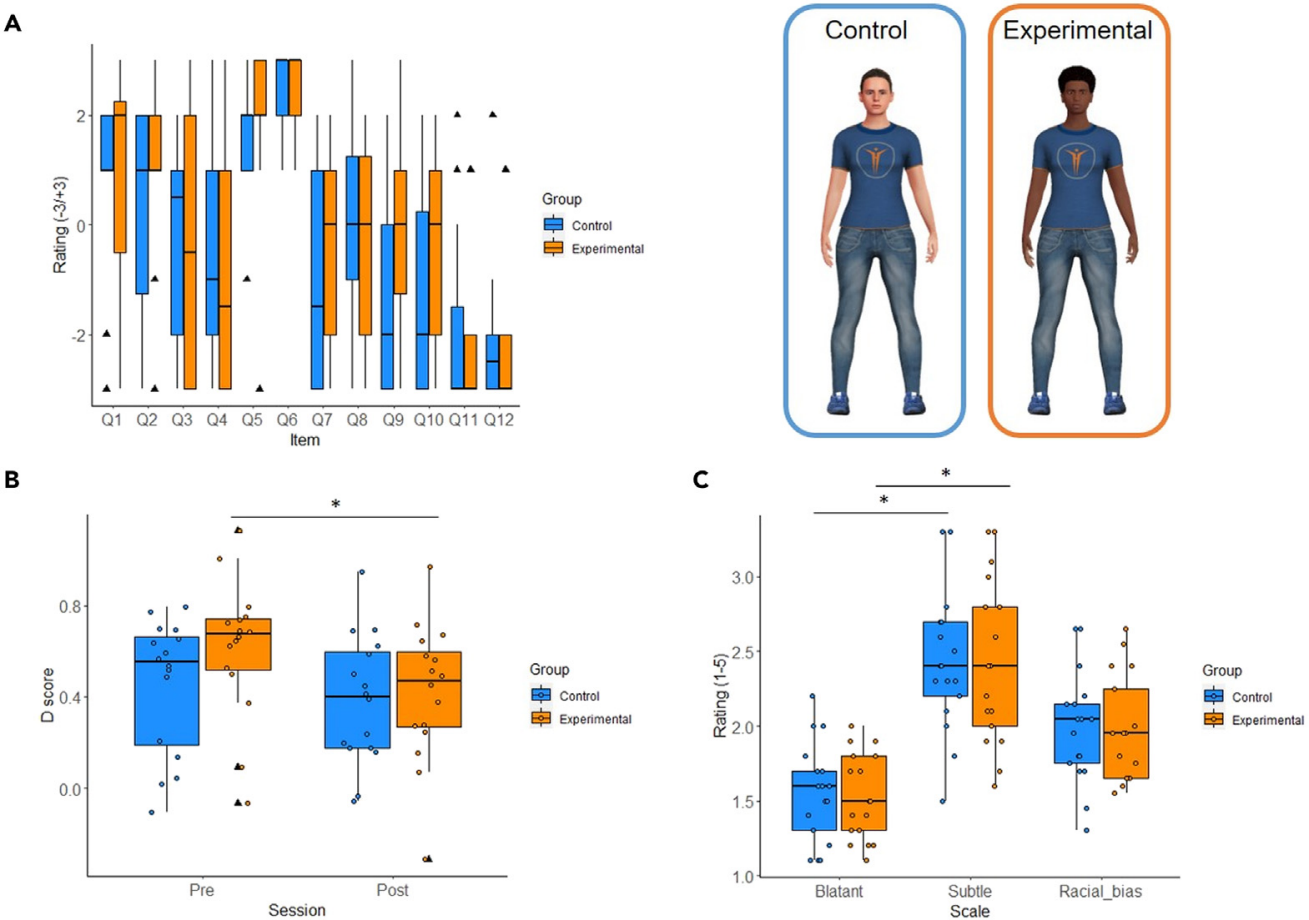
Behavioral results (A) Embodiment questionnaire ratings in the illusion (Q1–Q6) and the control (Q7–Q12) statements (−3/+3 scale). (B) Racial IAT scores in the two sessions (pre-embodiment, post-embodiment). (C) Explicit bias ratings on three scales—Blatant prejudice, Subtle prejudice, Overall Racial bias score (1–5 scale). The hinges of the box represent the first and the third quartile, with the line in the middle of the box representing the median and the bottom and top whiskers representing the smallest and the largest value no further than 1.5∗IQR from the lower and the upper hinges, respectively. The black triangles represent the outliers; the smaller dots represent individual values (panels B, C). ∗ = significant difference.

As presented in Figure 2A, the median scores in the questionnaire statements that refer to the feeling of owning the virtual body (Q1, Q2) and to the sense of agency over its movements (Q5, Q6) were over 1, whereas the median scores in all control statements were equal or less than 0 in both groups. Together, this suggests that both groups of participants experienced the key aspects of embodiment. The median scores in Q3 and Q4 that refer to the ownership of the avatar’s reflection in the mirror and to the identification with its appearance were close to zero or negative in both groups.

The results of the embodiment questionnaire analysis (see details in Table 1) showed that, in the between-group comparisons of the illusion statements (Q1-Q6), the scores were not significantly different between the groups in any of the statements. As regards the within-group comparisons of the illusion statements with the corresponding control statements, the pattern of significant differences was somewhat different between the groups. Specifically, the scores were significantly different in the control group in Q1 versus Q7 but not in Q2 versus Q8, whereas in the experimental group the differences in scores in both these pairs of statements were borderline non-significant; furthermore, the scores in Q3 were significantly higher than in its control item Q10 only in the experimental group, despite being negative. Instead, the groups had a comparable pattern of differences (illusion > control) in the two agency statements and the respective control ones and a comparable non-significant difference in Q4 versus Q10.

**Table 1:** Descriptive and tests statistics for the embodiment questionnaire analysis

Item N	Group (mean±SEM)		Between-group comparisons	Within-group comparisons		
	Control	Experimental	Experimental Versus Control	Items N	Control	Experimental
Q1	0.94±0.36	0.94±0.55	W = 102, p = 0.32	Q1 vs Q7	V = 8, p = 0.005	V = 27, p = 0.06
Q2	0.25±0.49	1.06±0.39	W = 98, p = 0.25	Q2 vs Q8	V = 49, p = 0.85	V = 24, p = 0.08
Q3	−0.31±0.45	-0.44±0.58	W = 131.5, p = 0.91	Q3 vs Q9	V = 10.5, p = 0.048	V = 77, p = 0.66
Q4	−0.38±0.49	-0.81±0.54	W = 149, p = 0.43	Q4 vs Q10	V = 22, p = 0.10	V = 40, p = 0.56
Q5	1.69±0.24	2.00±0.38	W = 88, p = 0.11	Q5 vs Q11	V = 0, p < 0.001	V = 1, p < 0.001
Q6	2.56±0.16	2.62±0.16	W = 120.5, p = 0.75	Q6 vs Q12	V = 0, p < 0.001	V = 0, p < 0.001
Q7	−0.88±0.49	−0.38±0.48	—	—	—	—
Q8	0.12±0.45	−0.38±0.48	—	—	—	—
Q9	−1.31±0.47	0.00±0.48	—	—	—	—
Q10	−1.31±0.49	−0.44±0.42	—	—	—	—
Q11	−1.88±0.45	−2.50±0.26	—	—	—	—
Q12	−2.19±0.32	−2.50±0.26	—	—	—	—

### Racial IAT

The d scores obtained in the racial IAT were compared between groups at baseline using an independent samples t test and within each group between two time points (baseline and post-embodiment) using a paired t test (see STAR methods for details).

The implicit racial bias (see Figure 2B) was not significantly different between groups at baseline (pre-embodiment): t = −1.58, p = 0.13; experimental group mean ± SEM = 0.62 ± 0.07; control group mean ± SEM = 0.45 ± 0.07. As regards the changes in the implicit racial bias between the two time points (pre- versus post-embodiment), it was significantly reduced in post-embodiment compared with pre-embodiment only in the experimental group (t = 2.22, p = 0.04, d = 0.67, post-embodiment mean ± SEM = 0.42 ± 0.08) but not in the control group (t = 1.02, p = 0.32, post-embodiment mean ± SEM = 0.38 ± 0.07). To summarize, the groups had comparable implicit racial bias at baseline, whereas after embodiment it significantly decreased only in the experimental group.

### Explicit racial bias

The scores on the Blatant and Subtle prejudices scales, as well as the general explicit bias score, were compared between groups using Mann-Whitney U test. Within each group, the scores were compared between the two scales using Wilcoxon signed-rank test (see STAR methods for details).

Firstly, both the overall explicit racial bias score, computed as the average of the Blatant and the Subtle prejudice scales scores, and the scores in each of the two scales were below the scale midpoint in both groups (see Figure 2C). In details, the scale ranges from 1 to 5, i.e., from “minimum prejudice” to “maximum prejudice,” and the scores of the sample were the following (mean ± SEM): Experimental group—Overall bias: 1.97 ± 0.09; Blatant: 1.52 ± 0.07; Subtle: 2.42 ± 0.14; Control group—Overall bias: 1.99 ± 0.09; Blatant: 1.55 ± 0.09; Subtle: 2.42 ± 0.12.

In addition, the scores were not significantly different between groups in any of the scales (Overall: U = 158, p = 0.65, Blatant: U = 149, p = 0.89, Subtle: U = 148.5, p = 0.90). Within each group, the scores on the Blatant prejudice scale were significantly lower compared with the Subtle prejudice (Experimental group: V = 1, p < 0.001, r = −0.72; Control group: V = 0, p < 0.001, r = −0.72).

To summarize, the two groups showed comparable levels of the explicit racial bias, which was on average low, with significantly higher subtle prejudice compared with the blatant one.

### Sentence reading task (EEG)

#### N400

**Figure 3:**
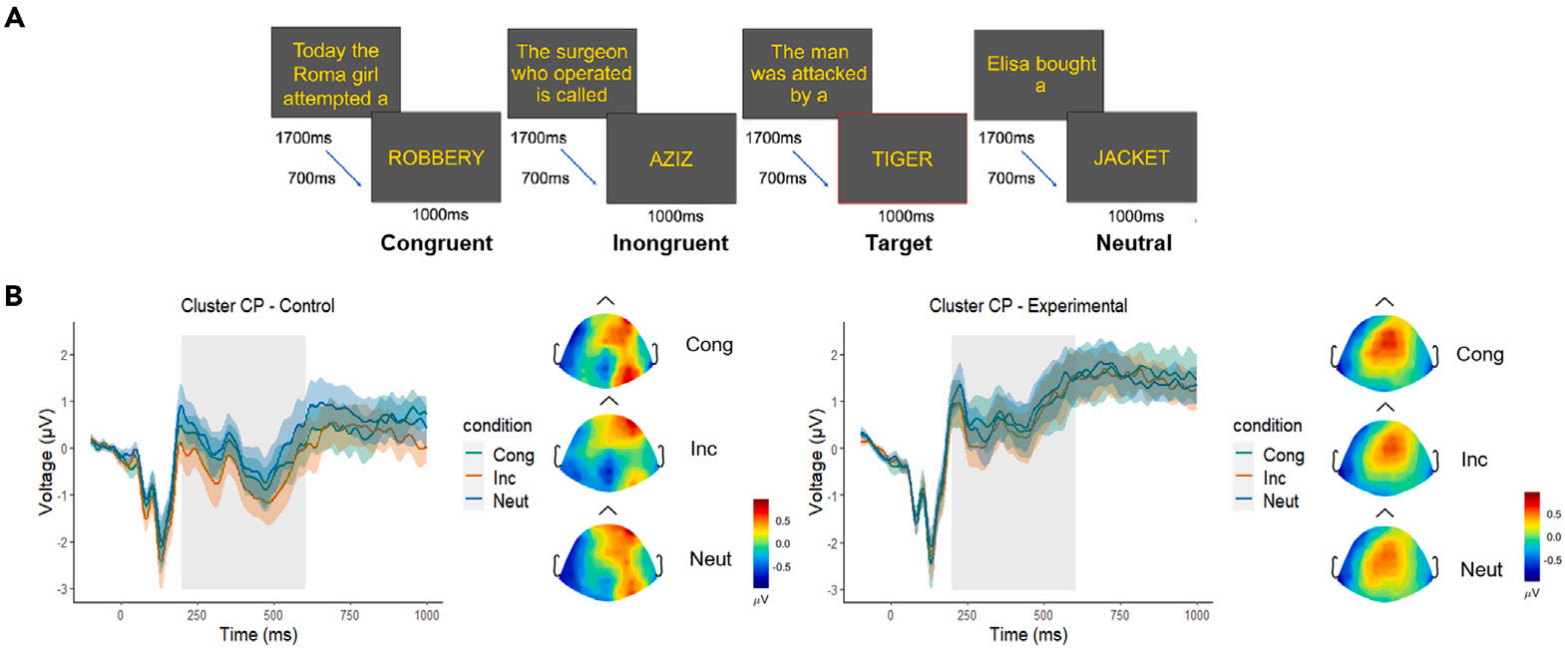
Sentence reading task and results in the time domain (ERP time series with standard errors) (A) Trial timeline and example of the stimuli (task, stimuli, and image adapted from^25^). (B) Results for the N400 at the centroparietal cluster and the topographical voltage distribution in each of the conditions; results of the control group are presented in the left panel, and the results of the experimental group are presented in the right panel. The gray rectangle highlights the interval of interest considered for statistical analysis. Cong, Congruent condition; Inc, Incongruent condition; Neut, Neutral condition; CP, centroparietal cluster.

The 2x3 ANOVA performed for the mean N400 amplitude in the 200–600 ms time window at the centroparietal cluster of electrodes showed a significant main effect of Condition [F(2, 60) = 4.37, p = 0.01, gη^2^ = 0.011], but no significant interaction [F(2, 60) = 0.83, p = 0.44, gη^2^ = 0.002], nor a significant main effect of Group [F(1, 30) = 3.36, p = 0.08, gη^2^ = 0.09]. Post-hoc paired comparisons for Condition showed that the N400 amplitude in the Incongruent condition (mean amplitude = 0.008 μV) was significantly larger compared with the one in Congruent (p = 0.03; mean amplitude = 0.26 μV) and Neutral conditions (p < 0.001; mean amplitude = 0.40 μV), which in turn were not significantly different between each other (p = 0.15). In other words, in both groups, the N400 amplitude was significantly larger in the Incongruent condition, compared with the Congruent and Neutral conditions. The results are presented in Figure 3B; an example of experimental stimuli and trial timeline are presented in Figure 3A.

#### P300

**Figure 4:**
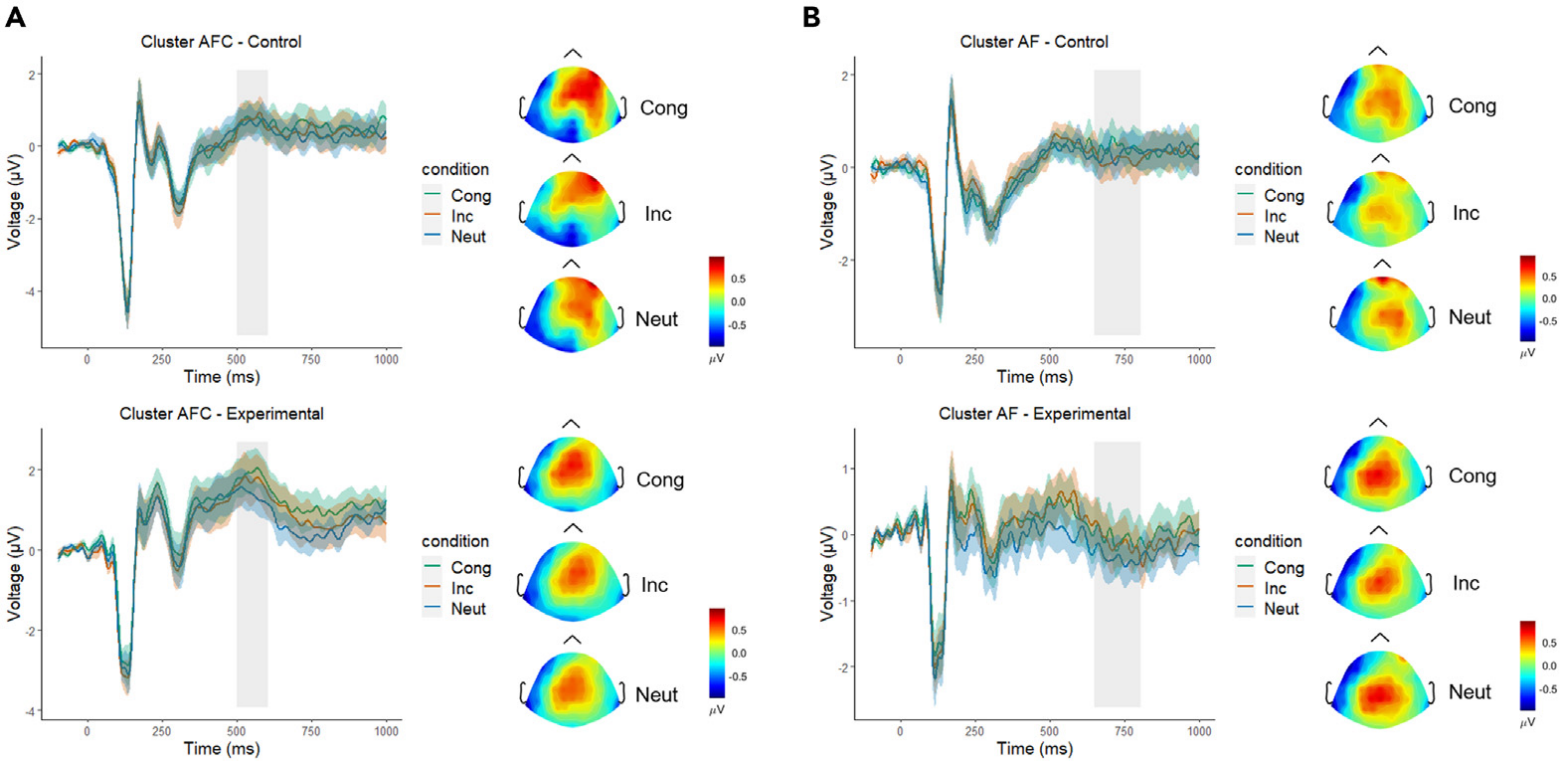
Results of the sentence reading task in the time domain (ERP time series with standard errors) for the P300 and the frontal positivity, with the respective topographical voltage distribution in each of the conditions (A) P300 at the fronto-central cluster. (B) Frontal positivity at the anterior-frontal cluster. Results of the control group are presented in the top panels, and the results of the experimental group are presented in the bottom panels. The grey rectangle highlights the interval of interest considered for statistical analysis. Cong = Congruent condition; Inc = Incongruent condition; Neut = Neutral condition; AFC = fronto-central cluster; AF = anterior-frontal cluster.

The 2x3 ANOVA of the P300 amplitude at the 500–600 ms time window at the fronto-central cluster (F1, F2, Fz, FCz) showed no significant effects: Group x Condition: F(2, 60) = 1.41, p = 0.25, gη^2^ = 0.003; Group: F(1, 30) = 2.51, p = 0.12, gη^2^ = 0.07; Condition: F(2, 60) = 2.23, p = 0.12, gη^2^ = 0.005. The results are presented in Figure 4A.

#### Frontal positivity

The 2x3 ANOVA of the frontal positivity amplitude at the 650–800 ms time window at the anterior-frontal cluster (AF3, AF4) showed no significant effects: Group x Condition: F(2, 60) = 1.12, p = 0.33, gη^2^ = 0.004; Group: F(1, 30) = 1.07, p = 0.31, gη^2^ = 0.03; Condition: F(2, 60) = 1.55, p = 0.22, gη^2^ = 0.005. The results are presented in Figure 4B.

#### Behavioral task

The mean accuracy in detecting the target stimuli, i.e., the animal names, of the sample was high (98.97 ± 0.16%), and it was not significantly different between the groups (t = −0.42, p = 0.68, Experimental group: mean ± SEM = 98.89 ± 0.23; Control group: mean ± SEM = 99.03 ± 0.25). This suggests that the attentive state of the participants was high throughout the task.

## Discussion

The present study aimed at detecting the neurophysiological signature of implicit racial bias modulation induced by embodiment of racially out-group virtual avatar. The N400 component of the ERPs was analyzed for terminal words of sentences that were congruent, incongruent, or neutral with respect to negative racial and ethnic stereotypes. In addition, the implicit racial bias was evaluated behaviorally with the racial IAT. Both measures were collected after virtual embodiment of an out-group (experimental group), or an in-group (control group) avatar, and compared between the groups. Furthermore, the racial IAT was administered before embodiment in a separate session to obtain a baseline measure of implicit racial bias and compare it with the post-embodiment level.

The results showed that, firstly, the virtual embodiment was comparable for the out-group and the in-group avatars. Secondly, the implicit racial bias was behaviorally reduced after the virtual embodiment, as compared with the pre-embodiment baseline, only in the experimental group, i.e., after the embodiment of a racially out-group avatar. It was not significantly different from the baseline in the group that embodied an in-group avatar. Crucially, in both groups, the N400 was present for the sentences that violated negative racial stereotypes, compared with those congruent or neutral with respect to such stereotypes. In other words, the first neurophysiological evidence of its kind suggested that embodying a racially out-group avatar did not affect the negative stereotypical response related to other-race people.

### Virtual embodiment

The fact that, in the present study, the avatar was embodied regardless of its appearance (skin color) is in line with the literature both on the embodiment of other-race avatars and, in general, on the embodiment of avatars with appearance that differ from participants’ own (for reviews see^8^^,^^27^). This is a consistent finding across multiple studies, and its explanation might lie in the nature of the body-ownership illusions and in the sense of immersion and presence provided by the IVR. As described in the introduction, a fake/virtual body, or a body part, is embodied whenever it is human-shaped, located in an anatomically plausible position, and receives a synchronous multisensory stimulation, such as visuomotor or visuotactile one.^7^ Visuomotor stimulation (the synchrony between the participant’s movements and those of the fake body), which is easy to achieve in the IVR, provides a strong feeling of agency over the movements of the fake body and thus acts as an additional cue for embodiment. Finally, the IVR allows presenting a virtual avatar from the 1PP, as if the avatar substitutes participant’s own body and participant observes the world from the perspective of this body. Combined with a strong sense of presence in the virtual environment,^28^ these multisensory cues create a strong feeling of embodiment, overriding the discrepancies between the avatar’s and participant’s appearance. This is a crucial point for the aims of the present study because comparable embodiment between the groups of participants, i.e., between the out-group and the in-group embodiment, allows interpreting the results in terms of the effects of embodiment, rather than a function of its strength *per se*.

Another point worth discussing is the virtual scenario employed in the present study. In particular, the embodiment procedure was rather simple and, importantly, did not contain any social context or interaction. In other words, participant did see a virtual body that had dark or light skin color from the 1PP and performed some simple movements, but no other virtual characters were present in the scene and none of the instructions, nor the embodiment questionnaire items, contained any reference to race or social situations. This makes the embodiment procedure implicit with respect to the racial bias and therefore allows interpreting the results purely as effects of the embodiment of an out-group body. Indeed, only one previous study on the racial bias^12^ used a similar “neutral” embodiment procedure, while other studies employed different variations of virtual scenarios where participant’s avatar either interacted with other virtual characters^10^^,^^13^^,^^29^ or other virtual characters were present.^9^^,^^30^^,^^31^^,^^32^

Taken together, the socially neutral virtual scenario and the comparable levels of embodiment in the two groups of participants allow treating the analyzed implicit and neurophysiological indices of the racial bias as directly related to the embodiment effects.

### Implicit and explicit racial bias

The reduction of the implicit racial bias that was observed after the embodiment of an out-group avatar, but not of an in-group one, is consistent with most of the previously reported findings (e.g.,^9^^,^^10^^,^^13^^,^^29^^,^^33^; see^8^). Two previous studies that showed an opposite pattern—an increase in the implicit racial bias after the out-group embodiment—included the negative/stressful social context in the virtual scenarios, which could explain the inverse effects that they observed.^30^^,^^34^ Therefore, the social context is an important factor to consider, especially if one aims to use the virtual embodiment of out-group avatars as a tool for mitigating implicit biases. However, because it was not the aim of the present study, our virtual scenario was neutral and context-free (unlike the majority of the previous studies, as described earlier). Our results, therefore, suggest that the sole feeling of owning out-group body and controlling its movements can affect the racial bias at the implicit level. This is an important point to consider both from theoretical and practical perspectives. It further stresses the key role of body ownership and body-related information in higher-level cognitive processes and consciousness as a whole and, in particular, it highlights how perceiving the self and the world from “the inside” of one’s body shapes every conscious experience.^35^^,^^36^

As regards the possible interpretation of this effect, one existing explanation^10^ seems to be the most prominent. In particular, it has been suggested that embodying an out-group avatar alters the individual statistical associations between the categories included in the IAT.^10^ Initially, the implicit racial bias against Other-Race people is represented by a stronger socially learned association between the categories “White, positive” and “Other-Race (e.g., Black), negative” than between the opposite categories. Then, during the embodiment of an out-group avatar, another association is added, which is “Self, Black” (because embodiment means the feeling that the body one sees belongs to oneself, so the skin color of that body must belong to oneself, too). Consequently, this new association weighs into the associations between the IAT categories, for example, through another learned association—“Self, positive.” Therefore, the association “Black, negative” might be disrupted by the embodiment-induced feeling that “I can be Black,” combined with the pre-existing association between “Self” and “positive,” thus reducing the implicit racial bias.^10^^,^^11^ It is necessary to note that this explanation remains speculative because neither the original study that suggested it,^10^ nor other embodiment studies on the topic, nor the present study directly tested the implicit bias of the categories “Self, positive” and “Other, negative.”^37^

Crucially, however, in the present study, the racial IAT served mainly as a control measure in order to confirm that the employed embodiment procedure and scenario evoked the effects similar to those previously reported. As discussed in the introduction, the IAT is a key measure of the implicit biases, but it probes only one aspect of the phenomenon (the evaluative component of the bias) and does not provide evidence into the extent, or the neural correlates, of the embodiment effects on other components of the bias.

Another control measure was the explicit racial bias that was evaluated with a questionnaire and represented as a general score that was further divided in Blatant and Subtle prejudices. We did not expect to observe high, or even above-average, levels of explicit racial prejudices in our sample, in line with,^26^ which was, indeed, that case. Moreover, we did not observe significant differences in the explicit racial bias between the groups. It might suggest that the explicit racial bias was not influenced by the skin color of the embodied avatar, which would be in line with^38^ that demonstrated that, in general, the interventions for mitigating the racial bias do not affect the explicit bias, and in line with^30^ that showed similar absence of significant differences after embodiment of Black versus White avatar. However, this conclusion should be considered with caution because the explicit racial bias was evaluated only post-embodiment, and it is impossible to discuss its embodiment-related changes without a baseline measure.

### Neurophysiological results

As regards the key measure of the present study, there were no significant of between-group differences (i.e., embodiment effects) in the N400 component of the ERPs in response to the stimuli that violated negative racial stereotypes. It is the first attempt to add neurophysiological evidence of the embodiment effects on the implicit biases to the existing behavioral data.

The N400 is a well-established neurophysiological correlate of implicit social biases, including sex bias^24^^,^^39^ and the racial bias.^22^^,^^25^ In general, it has been described as being related to the difficulty in integrating the incoming semantic information with the previous knowledge of the world.^21^ Within this logic, the N400 is observed for the stimuli that go against the pre-existing implicit stereotypes about different races and ethnicities and, in particular, for those that convey socially positive information and thus go against the implicitly strong association between non-White ethnicities and negative assumptions.

Here, we observed the N400 for sentences that violated the negative racial stereotypes, compared with the sentences that did not violate them or were racially neutral. This result replicates the previous findings and further confirms the relation between the N400 and the implicit racial bias.^22^^,^^25^^,^^26^ Contrary to our main hypothesis, embodiment of a racially out-group avatar did not modulate the N400, i.e., at the neurophysiological level, we did not observe a reduction of the implicit racial bias following the out-group embodiment that we observed at the level of implicit associations (in the racial IAT). There might be several factors contributing to this result.

Firstly, it is possible that the dissociation between embodiment effects at the implicit and the neurophysiological levels is driven by the fact that the IAT and the N400 task concern different components of the racial bias. In detail, the racial IAT concerns the evaluative component of the racial bias (i.e., the evaluative attitudes in the attribution of Black and White categories to the “pleasant” and “unpleasant” categories). In turn, sentences in the N400 task reflect the cognitive component of the racial bias that implies the learned associations (racial stereotypes) that are independent from the evaluative attitudes. Indeed, the dissociation of these two components of the racial bias has been demonstrated at the neural level in the neuroimaging studies that showed the activation of distinct neural networks related to affective and semantic processes, respectively.^16^^,^^17^ In the context of the present study, the results suggest that embodying an out-group avatar affected the evaluative component of the racial bias, as represented by the IAT, but not the cognitive component that was represented by the N400. Indeed, the possible speculative interpretation of the embodiment effects of the IAT presented earlier is based on the shift in the attribution of the concepts (Black, White) to the evaluative/emotionally relevant categories (“positive” and “negative”) that occurs due to the changes in the concept of the Self through embodiment. The same embodiment procedure seems to not affect the cognitive (semantic) component of the racial bias, thus not affecting the N400 related to the semantic violation of racial stereotypes. This might occur because embodiment does not change the learned information that is already present, because this information is treated separately from the evaluative affective component of the racial bias and cannot be influenced by its changes.

In addition to this main interpretation, it is necessary to consider the differences in the stimuli in the racial IAT and the N400 task. Differently from the IAT and the embodiment procedure per se that included a two-level racial category (Black and White), the N400 task contained a set of stimuli (sentences) with the racial stereotypes about multiple ethnic groups. In fact, it is known that the explicit interventions that successfully reduce implicit bias against Black people do not induce a similar reduction for the biases against other non-White ethnic groups,^38^ and the previous embodiment studies on the racial bias always included the same two categories in the embodiment and the IAT procedures. We chose to use the set of stimuli with multiple ethnic stereotypes because it had been previously validated for the relevant population^25^ and employed in a similar context,^26^ thus allowing to compare our results with existing data. Our results, however, further confirmed that the implicit bias modulations are bias-specific and do not generalize. Interestingly, differently from our study, the study by Brusa et al.^26^ used this set of stimuli and did show the reduction of the N400 after an explicit manipulation of the racial bias. However, that manipulation of the racial bias contained positive information about multiple ethnic groups, i.e., it corresponded with the content of the stimuli, which might suggest that the reduction of the racial bias was not generalized but rather concerned several groups targeted at once. In addition, it might have affected the N400 because it directly presented information (videos of people of different ethnicities engaged in “socially positive” activities) that could temporarily update the existing stereotype-related information, whereas the embodiment procedure, as discussed earlier, was implicit in nature and did not contain any socially relevant information.

It is necessary to note that, contrary to the hypotheses, we did not obtain any significant effects of condition (stimulus type) on the frontocentral P300 and the frontal positivity. We expected to replicate the results of the previous studies^25^^,^^26^ that showed larger P300 for the stimuli congruent with the negative racial stereotypes, and larger frontal positivity for the congruent and incongruent stimuli, i.e., those referring to the racial stereotypes, compared with the neutral ones. In addition, these effects were not expected to be modulated by the embodiment condition. We confirmed the latter result, i.e., no significant effect of group (embodiment), which can be considered as the most relevant for the present study. It is unclear, however, why the effects of stimulus type were not significant for these two components but, because they do not directly reflect the implicit racial bias, it does not impact the main result of the study.

To summarize, our results suggest that the embodiment of a racially out-group avatar did not affect the neurophysiological index of the cognitive (semantic) component of the racial bias, whereas it did affect its evaluative (affective) component. Considering that explicitly targeting the cognitive aspect of the racial bias, for example, by presenting racially positive information, is known to be effective only in the short term,^1^^,^^4^ and trying to affect it implicitly, such as through embodiment, seems to not affect at least the cognitive component of the process, developing other interventions remains a crucial open issue. Possibly, such interventions could combine the implicit approach with presentation of the explicit information—for example, if racially positive information, or behavior, would be integrated within the out-group embodiment, it might have more sustained effects on the stereotypes, i.e., the cognitive aspect of the racial bias.

### Conclusions

The present study investigated the behavioral and neurophysiological effects of embodying a racially out-group virtual body on the implicit racial bias against that group. The results showed that embodiment of an out-group avatar was followed by a reduction of the implicit racial bias, as represented by the racial IAT, but not of the neurophysiological index of the cognitive component of the racial bias, represented by the N400 in response to the violation of negative racial stereotypes. Together, the results provide the first evidence that the embodiment can affect the evaluative component of the implicit biases but not their cognitive component.

This might be considered in the development of novel interventions for targeting the implicit biases, for example, by combining the embodiment of out-group avatars with exposure to race-positive information or behavior of the embodied avatar and/or other virtual characters in the scene. To this end, the semantic N400 might be further used as the neurophysiological index of the stereotyping that should be considered in combination with the evaluative index of the bias (the implicit attitudes).

Another point to address in the future studies would be to investigate whether these results can be generalized to other types of implicit biases, such as the gender, age, or weight biases. The behavioral evidence suggests that it might be the case for the IAT^8^; however, no study to date directly compared the embodiment effects on different implicit biases with a set of identical measures, which might include the IAT and the N400 in order to probe different components and levels of the process.

### Limitations of the study

It is necessary to point out several limitations of the present study. The first one concerns the control conditions in the embodiment procedure. In particular, having two conditions that varied in the skin color of the avatar, but included both the 1PP and the visuomotor synchrony, does not allow to disentangle the effects of embodiment and the possible effects of merely being exposed to the virtual environment and the avatar. Although our experimental conditions match those most commonly used in the literature on the topic (see^8^ for review), the study would have benefitted from introducing a condition that would serve as control for the exposure effects separately from embodiment. However, identifying such condition is not straightforward because the two most reasonable options—the 1PP condition with visuomotor asynchrony and the 3PP condition—are not fully suitable for this purpose. In detail, the condition with visuomotor asynchrony in the 1PP would result in the absence of the sense of agency but not necessarily in the full absence of body ownership. This is due to the fact that the 1PP in IVR is among the strongest cues for embodiment/ownership,^5^ and therefore, the visuomotor asynchrony might reduce ownership but not abolish it. This is the case for the visuotactile stimulation in the 1PP,^5^ and although the visuomotor (a)synchrony is potentially a stronger cue than the visuotactile one, it is reasonable to expect similar results (e.g., in^40^ ownership was reduced but still present to an extent in the condition of visuomotor asynchrony). One condition that would allow abolishing ownership/embodiment entirely is the condition of the 3PP, but it would not allow controlling for the effects of exposure to a virtual body in the 1PP. Therefore, designing an optimal control condition for the exposure effects in the VR embodiment studies remains an open issue.

Another limitation concerns the lack of baseline measures of the N400 and explicit racial bias. As regards the N400, the baseline measures were not collected because the same validated set of stimuli could not be administered twice within the same participants. Therefore, to potentially obtain the N400 measures at several time points in a within-study design, another comparable set of stimuli should be created. As regards the explicit racial bias, it was not possible to collect this measure at baseline without introducing possible effects on participants’ awareness about the study purpose. Although the absence of baseline data on the explicit bias does not allow stating that the groups were comparable in the explicit bias levels before participating in the study, we expected uniformly low levels of explicit bias in our sample, consistently with^25^^,^^26^ because our sample and the samples in these studies were comparable in the sociodemographic characteristics. However, in order to make conclusions about embodiment effects on the explicit bias, future studies should consider including a baseline measure of the explicit bias, although it would be problematic to control for its effects on the awareness of the study purposes.

Finally, the non-significant results of the between-group analysis of the N400 do not indicate the absence of between-group difference, but rather the absence of evidence in favor of such difference. Therefore, this result might be driven not only by the true non-significant difference but also by other factors, such as insufficient statistical power, despite the sample size required *a priori* being reached. To address this point, future studies might use Bayesian hypothesis testing.

## STAR★Methods

### Key resources table

**Table:** 

REAGENT or RESOURCE	SOURCE	IDENTIFIER
**Deposited data**		

Behavioral and EEG data of 34 human participants and analysis code	OSF repository	https://osf.io/tdhgn/?view_only=74fb972cda964ccbadc25ed1a64a9f77

**Software and algorithms**		

Unity (version 2021.3.4.f1)	Unity Technologies	https://unity.com/
Autodesk 3ds Max 2023 (Academic license)	Autodesk Inc.	https://www.autodesk.com/products/3ds-max/overview?term=1-YEAR&tab=subscription
MakeHuman (version 1.1.1)	The MakeHuman Team (2017)	http://www.makehumancommunity.org/
R (version 4.0.3)	R Core Team, 2022	https://www.R-project.org/
R Studio (2023.03.0 + 386)	R Studio Team, 2022	https://www.R-project.org/
MATLAB (version R2020a)	The Mathworks Inc.	https://www.mathworks.com/products/matlab.html
OpenSesame (version 3.3.5)	Mathôt, S., Schreij, D., & Theeuwes, J., 2012	https://osdoc.cogsci.nl/

### Resource availability

#### Lead contact

Further information and requests should be directed to and will be fulfilled by the lead contact, Maria Pyasik (maria.pyasik@unito.it).

#### Materials availability

The study did not generate new unique materials.

### Experimental model and study participant details

#### Participants

Thirty-four healthy Caucasian volunteers with normal or corrected-to-normal vision and no history of neurological or psychiatric disease gave written informed consent for participating in the study. Before the first experimental session, they were blindly assigned either to the Experimental (out-group), or the Control (in-group) group according to the skin color of the avatar, with gender being the only factor considered in the group assignment to obtain gander-balanced groups. Final sample included 32 participants (mean age ±SD = 23.8 ± 4.3; mean education ±SD = 17 ± 2.3) – 16 in the Experimental group (9 F, 7 M), and 16 in the Control group (10 F; 6 M); 2 participants were excluded from the final analysis due to the fact that EEG artifacts were present in more than 25% of the trials.

The study was approved by the Bioethical Committee of the University of Turin (protocol number 0233499).

### Method details

#### Experimental setup, materials and procedure

The experiment consisted of two sessions administered at least one week apart (see Figure 1A for the experiment task; Figure 1B for the timeline of session 2). During the first session, participants’ demographical data was collected and the pre-embodiment (i.e., baseline) racial IAT was administered. In the second session, after being fitted with the EEG cap, participants firstly experienced embodiment of an out-group (Experimental), or in-group (Control) virtual avatar, which was followed by an embodiment questionnaire, the sentence reading task with the EEG recording, post-embodiment racial IAT and a questionnaire evaluating explicit racial bias. The first session lasted approximately 15 min, while the second one was 1.5–2 h long. All experimental tasks and measures are described in detail below.

##### Virtual embodiment

The virtual scenario was designed using 3D Studio Max 2023 (Autodesk, Inc.) and implemented in Unity 2021 game software environment (http://unity.com). The virtual environment consisted of a virtual room with office furniture and a large mirror. A virtual avatar (1:1) was standing in front of the mirror. The avatar was gender-matched with the participant and either dark-skinned (Experimental condition) or light-skinned (Control condition). The avatars were created with MakeHuman software (http://www.makehumancommunity.org/).

Participant observed the virtual scenario through an Oculus Rift S head-mounted display [HMD, www.oculus.com, with a 110° field-of-view (diagonal FOV) and a resolution of 2560 × 1440] and controlled the avatar’s movements via the Oculus Rift S handheld controllers. Once the HMD was calibrated, participant, who was standing on her feet and holding the VR controllers in both hands, was immersed in the virtual scenario (see Figure 1B). She observed the virtual scene from the 1PP, i.e., from the avatar’s point of view and as if the avatar replaced her real body, and could freely move her hands, arms, torso, and head; these movements were synchronized with the movements of the avatar via tracking of the head-mounted display and handheld game controllers, thus providing the visuomotor synchrony.

The embodiment phase lasted approximately 5 min and began with the familiarization with the virtual environment – participant was asked to look around the virtual room, as well as down at the avatar (as if looking down at her own body) and in the mirror. After that, the instructions were provided about the constraints of the movements; in particular, participant was asked to not move her feet, as they were not tracked in the VR, and apart from that, freely perform any movements with the head, torso, arms, wrists and bend the legs. Next, participant was required to focus on the avatar, alternating between looking down at the body and looking in the mirror, and moved freely for approximately 2 min. This was followed by a set of instructions about the movements to perform that was identical for all participants (approximately 2 min). It included arm movements, wrist movements, tracing geometric shapes with each arm in the air in front of the participant and tracing their name. Finally, participant was asked to perform some more free movements for another minute and then to stand still.

At the end of the procedure, the embodiment questionnaire was administered. It consisted of 12 statements that described different aspects of embodiment (ownership, self-location, identification with avatar’s appearance, agency of the avatar’s movements) and the respective control statements, and was adapted from previous studies^10^^,^^40^; see Table S1. Participant was asked to rate the level of agreement with each statement of a −3 to +3 Likert scale, where −3 indicated complete disagreement and +3 complete agreement. The statements were administered verbally by the experimenter in randomized order and the responses were given verbally. After that, the virtual scenario was stopped and the HMD was taken off.

##### Sentence reading task

The task and stimuli were identical to those in the studies by Brusa et al.^25^^,^^26^ It consisted of reading sentences that were congruent, incongruent, or neutral with negative stereotypes with several ethnic groups (Eastern Europeans, Arabs, Africans, Asians, South Americans, Roma). The stimuli were balanced for the occurrence of those ethnic groups and were validated for the specific population (Italian university students).

Immediately after the conclusion of the embodiment procedure, participant was seated at a table in front of a computer screen (100 cm away from the participant). The instructions were presented on the screen and described the fictitious task: participant was required to read the sentences that would be presented on the screen, one by one, and press the response button when the terminal word of a sentence is a name of an animal. The task was therefore implicit, since the real aim of the task was not stated, and participant was not required to make any judgments about the content of the sentences.

The stimuli were presented with OpenSesame software.^41^ Each sentence was presented for 1700 ms; it was arranged in the center of the screen in a maximum of three short rows. It was followed by an ISI of 700 ms, after which the terminal word was presented for 1000 ms in the center of the screen in the uppercase letters, followed by the ITI of 1200 ms that contained a fixation cross in the center of the screen. The terminal words were balanced across conditions in terms of word length, frequency of use, word category, concreteness, and luminance. The font color of the stimuli was yellow on black background (see Figure 3A for stimulus example and trial timeline).

After a brief training block, each condition (congruent, incongruent, neutral) consisted of 95 sentences, while the target stimuli for the fictitious task included 24 sentences that contained the names of animals and had a lexical structure identical to the main stimuli. A total of 309 sentences were presented over 8 blocks (approximate duration – 2 min 40 s each) with randomized order within each block and the counterbalanced order of blocks across participants. Participants were instructed to alternate the hand used to press the response button between the blocks.

The EEG was recorded continuously during the sentence reading task with the event marks corresponding to the beginning of the terminal word presentation (see details below), and the behavioral responses were recorded for the target words in order to control participant’s accuracy and attentive state. The sentence reading task was immediately followed by the racial IAT and the questionnaire to evaluate the implicit and the explicit racial bias, respectively.

##### Racial IAT

The implicit racial bias was measured twice – during the first session (pre-embodiment, i.e., baseline) and during the second session following the virtual embodiment and the sentence reading task (post-embodiment), using the racial IAT.^14^ The IAT followed a standard procedure^14^ and was administered with the Inquisit software (https://www.millisecond.com/) on the same computer screen with the same response buttons across the two sessions.

The racial IAT required the participants to rapidly categorize images of faces (White, Black) and words (positive, negative) by pressing one of the two buttons with left or right hand. In detail, the face stimuli included 10 images of White faces and 10 images of Black faces taken from the stimuli dataset of Project Implicit’s Race IAT (osf.io/y9hiq); in turn, the word stimuli included 10 Italian words belonging to the category “positive” and 10 belonging to the category “negative”. The task began by the presentation of the introductory instructions on the screen that described the main purpose of the task (categorization of stimuli, i.e., White vs. Black faces and positive vs. negative words), along with a table that contained all the stimuli. This was followed by 7 blocks of trials, where the first 4 blocks had identical structure as the second 3 blocks and the only differences between them were the mapping of the response categories (which was reversed in the second part of the blocks) and the amount of training blocks. In the first block, participant was presented only with the images of faces at the center of the screen and the words “White” and “Black” presented in the top left and the top right of the screen to remind participant of the categories. Participant was required to press one of the buttons to respond whether the presented face belonged to the category on the left or to the one on the right (e.g., Z = left, M = right). In the second block, the same procedure was repeated for the words, with each word stimulus presented in the center of the screen and the two categories (“positive” and “negative”) in the top left and the top right. In the third block, the categories were combined, and both faces and words were presented in the center of the screen (one stimulus per trial), while the category labels in the top left and right corners of the screen contained a pair of categories, one referring to the faces and one referring to the words (e.g., “White, positive” and “Black, negative”, or vice versa depending on the blocks sequence). These three blocks consisted of 20 trials each and were considered as practice blocks. Finally, the fourth block (i.e., critical block), was identical to the third block but contained 40 trials. Blocks 5 to 7 were identical to the first four blocks, except for the mapping of the response buttons and the pairing of the categories. In detail, in block 5, which contained single-concept categorization of the words, the response buttons were switched, i.e., M = left, Z = right. Then, in the two blocks (6 and 7) with the combined categories, the pairs of categories were inverted with respect to block 3 and 4 (e.g., “White, negative” and “Black, positive”). The entire task, therefore, included 5 practice blocks of 20 trials each and 2 critical blocks of 40 trials each.

The mapping of the buttons and the order of the blocks (“White, positive; Black, negative” and “White, negative; Black, positive”) were counterbalanced across participants. The task lasted approximately 5.5 min.

The implicit bias was represented by a d score that was calculated from the reaction times of categorizing differently grouped stimuli (i.e., “White, positive; Black, negative” and the opposite group) and factored in both the practice and the critical blocks.^14^ Higher d score represented stronger implicit racial bias, as it reflected slower reaction times and less accurate responses for the pairing of white faces and negative words, and black faces and positive words.

##### Explicit racial bias

The second session was concluded by the explicit racial bias evaluation that was performed with a Subtle and Blatant Prejudice Scale.^42^^,^^43^ This 20-item questionnaire evaluates the explicit prejudices against non-Italian immigrants in Italy. It consists of two subscales of 10 items that address blatant and subtle prejudices, respectively, and contain several reverse-score items each. The agreement with the items is expressed on a 5-point Likert scale; the mean score is then computed for each of the two scales, and the mean of the two scales represents the general prejudices score.

#### EEG recording and processing

The EEG signal was recorded using a *BrainAmp* amplifier (Brain Products GmbH, Munich, Germany) with 64 Ag/AgCl electrodes mounted in an elastic electrode cap, arranged according to the international 10–20% system.^44^ The vertical and horizontal electro-oculogram (EOG) was recorded from the electrodes placed on the outer canthus of the right eye and from under the right eye for the horizontal and the vertical EOG, respectively. The signal was recorded continuously at the sampling rate of 1000 Hz and the impedances were kept under 10 k Ω. The signal was referenced to the FCz electrode and re-referenced offline to the average, with the signal from the FCz added back to the recording.

The offline preprocessing of the continuous raw data included a band-pass filter (0.016–30 Hz), followed by the independent component analysis (ICA^45^) where the components related to blinks and eye movements were identified and removed (mean number of removed ICA components ±SD = 3.2 ± 1.5). The signal was then downsampled to 500 Hz and segmented into epochs of −100 to +1000 ms. The epochs were time-locked to the onset of the terminal word of each sentence and baseline corrected to the interval between −100 ms before the terminal word onset and its onset (0 ms). All epochs were visually inspected for the remaining eye-movement artifacts, and signals with amplitude exceeding −100/+100 μV were rejected [mean number of epochs rejected ±SD = 21.2 ± 15.3 out of the total of 309; per condition: Congruent: 6.6 ± 5.7, Incongruent: 7.3 ± 4.8, Neutral: 7.2 ± 5.8; no significant differences were present between the number of rejected epochs in each condition (F = 0.14, p = 0.89)].

The epochs were averaged for each condition (Congruent, Incongruent, Neutral). The epochs related to the target stimuli (animal names) were not included in the analysis, as these stimuli were used for the fictitious task and to control participant’s attention. The mean amplitude area for the N400 was analyzed in the 200–600 ms time window over the centroparietal cluster of electrodes (Cz, CPz, Pz). The time window of the N400 peak and the analyzed cluster were selected according to the literature.^21^ In addition, in line with Brusa et al.^25^^,^Brusa et al.,^26^ the mean amplitude area of P300 was analyzed in the 500–600 ms time window at a cluster of fronto-central electrodes (F1, F2, Fz, FCz), and the mean amplitude area of frontal positivity was analyzed in the 650–800 ms time window at a cluster of anterior-frontal electrodes (AF3, AF4).

Data preprocessing was performed using the Brainstorm toolbox for MATLAB (https://neuroimage.usc.edu/brainstorm/;^46^); ERP data extraction and analysis was performed in R Studio^47^

### Quantification and statistical analysis

Statistical analyses were performed with R software in R Studio.^47^

The sample size was based on the mean effect size reported in previous studies on the virtual embodiment and the racial bias^9^^,^^10^ and the EEG correlates of the racial bias in a between-group design^26^ (_p_η^2^ = 0.127). According to *a priori* power analysis, performed for the main outcome measures (EEG), for a 2x3 mixed effects ANOVA with Group (Experimental, Control) as between-subject factor and Condition (Congruent, Incongruent, Neutral stimuli) as within-subject factor, in order to achieve the power of 0.80 and the alpha level of 0.05, the required sample size was 28 participants (14 per group). We aimed at recruiting several participants over than number in order to account for potential dropouts and for participants excluded due to bad EEG signal.

#### Behavioral data

##### Embodiment questionnaire

the ratings for each of the illusion statements were compared with planned comparisons between the groups of participants using Mann-Whitney U test. In addition, each illusion statement was compared with the corresponding control statement within each group using Wilcoxon signed rank test.

##### Racial IAT

The d scores were analyzed with planned comparisons to, firstly, compare the baseline implicit racial bias between the two groups (pre-embodiment), and then compare it within each group for post-vs. pre-embodiment levels. These analyses were performed with unpaired or paired t tests, respectively.

##### Subtle and Blatant Prejudice Scale

The scores on each scale, as well as the general explicit bias score, were compared between groups using Mann-Whitney U test. Within each group, the scores were compared between the blatant and the subtle prejudice scales using Wilcoxon signed rank test.

#### EEG data

All ERP analyses were performed on mean amplitudes of the peaks.^48^ For each of the analyzed ERP components (N400, P300, frontal positivity), a 2x3 mixed effects ANOVA was performed with Group (Experimental, Control) as a between-subject factor and Condition (Congruent, Incongruent, Neutral) as a within-subject factor.

The behavioral responses in the sentence reading task were analyzed as the task accuracy (i.e., the number of correctly identified target stimuli, in %) and compared between the two groups using an independent samples t test.

Effect sizes were estimated using generalized eta squared (gη^2^) or Cohen’s d (for parametric tests), or Pearson’s correlation coefficient r (for nonparametric tests). All multiple comparisons were corrected with Holm-Bonferroni correction. Details about descriptive (i.e., means and SDs) and inferential statistics (i.e., mixed-effects ANOVA, Wilcoxon signed-rank test, Mann-Whitney U test, t test, p values and effect sizes) can be found in the results section, Table 1 and Figures 2, 3, and 4. Alpha was set at 0.05.

## Data Availability

• The full dataset with behavioral data and all raw and processed EEG data have been deposited in the OSF repository and is publicly available. URLs are listed in the key resources table. • All original code has been deposited in the OSF repository and is publicly available. URLs are listed in the key resources table. • Any additional information required to reanalyze the data reported in this paper is available from the lead contact upon request.

## Supplemental information

Table S1. Embodiment questionnaire; the agreement with each statement was rated on a -3 to +3 Likert scale where -3 indicated complete disagreement and +3 complete agreement
