# Supplementary material for: Behavioral and neurophysiological indices of the racial bias modulation after virtual embodiment in other-race body
Source: iScience. 2023 Sep 28;26(10):108085. doi: 10.1016/j.isci.2023.108085 (PMC10582573; doi:10.1016/j.isci.2023.108085)
Supplement: Table S1. Embodiment questionnaire; the agreement with each statement was rated on a -3 to +3 Likert scale where -3 indicated complete disagreement and +3 complete agreement [file mmc1.pdf]

iScience, Volume *26*

Supplemental information

Behavioral and neurophysiological indices of the racial bias modulation after virtual embodiment in other-race body

Maria Pyasik, Alice Mado Proverbio, and Lorenzo Pia

Table S1. Embodiment questionnaire; the agreement with each statement was rated on a -3 to +3 Likert scale where -3 indicated complete disagreement and +3 complete agreement.

Statement type	Illusion statements	Control statements
**Location**	Q1. There were moments in which it seemed that my body was located where the virtual body was located.	Q7. There were moments in which it seemed that I was outside of my body.
**Ownership**	Q2. There were moments in which it seemed that the virtual body that I saw when looking down was my own body.	Q8. There were moments in which it seemed that the virtual body belonged to someone else.
**Ownership (Mirror)**	Q3. There were moments in which it seemed that the virtual body that I saw reflected in the mirror was my own body.	Q9. There were moments in which it seemed that I had two bodies.
**Appearance**	Q4. There were moments in which it seemed that the virtual body resembled my own real body in terms of shape, skin color or other features.	Q10. There were moments in which it seemed that my body had disappeared.
**Agency**	Q5. There were moments in which it seemed that the movements of the virtual body were my movements.	Q11. There were moments in which I had the sensation of being dragged.
**Volition**	Q6. There were moments in which it seemed that I could control the movements of the virtual body.	Q12. There were moments in which it seemed that the movements of the virtual body were controlled by someone else.
